# The receptor-type protein tyrosine phosphatase CD45 promotes onset and severity of IL-1β–mediated autoinflammatory osteomyelitis

**DOI:** 10.1016/j.jbc.2021.101131

**Published:** 2021-08-27

**Authors:** Jarmila Kralova, Nataliia Pavliuchenko, Matej Fabisik, Kristyna Ilievova, Frantisek Spoutil, Jan Prochazka, Jana Pokorna, Radislav Sedlacek, Tomas Brdicka

**Affiliations:** 1 Laboratory of Leukocyte Signaling, Institute of Molecular Genetics of the Czech Academy of Sciences, Prague, Czech Republic; 2 Charles University, Faculty of Science, Prague, Czech Republic; 3 Czech Centre for Phenogenomics, Institute of Molecular Genetics of the Czech Academy of Sciences, Vestec, Czech Republic; 4 Laboratory of Transgenic Models of Diseases, Institute of Molecular Genetics of the Czech Academy of Sciences, Vestec, Czech Republic

**Keywords:** CD45, PTPRC, PSTPIP2, autoinflammation, chronic recurrent multifocal osteomyelitis, CMO, chronic multifocal osteomyelitis, FBS, fetal bovine serum, IL-1β, interleukin-1β, ITAM, immunoreceptor tyrosine-based activation motif, LPS, lipopolysaccharide, μCT, microcomputerized tomography, ROS, reactive oxygen species, SFK, Src-family kinase, SYK, spleen tyrosine kinase

## Abstract

A number of human autoinflammatory diseases manifest with severe inflammatory bone destruction. Mouse models of these diseases represent valuable tools that help us to understand molecular mechanisms triggering this bone autoinflammation. The *Pstpip2*^*cmo*^ mouse strain is among the best characterized of these; it harbors a mutation resulting in the loss of adaptor protein PSTPIP2 and development of autoinflammatory osteomyelitis. In *Pstpip2*^*cmo*^ mice, overproduction of interleukin-1β (IL-1β) and reactive oxygen species by neutrophil granulocytes leads to spontaneous inflammation of the bones and surrounding soft tissues. However, the upstream signaling events leading to this overproduction are poorly characterized. Here, we show that *Pstpip2*^*cmo*^ mice deficient in major regulator of Src-family kinases (SFKs) receptor-type protein tyrosine phosphatase CD45 display delayed onset and lower severity of the disease, while the development of autoinflammation is not affected by deficiencies in Toll-like receptor signaling. Our data also show deregulation of pro-IL-1β production by *Pstpip2*^*cmo*^ neutrophils that are attenuated by CD45 deficiency. These data suggest a role for SFKs in autoinflammation. Together with previously published work on the involvement of protein tyrosine kinase spleen tyrosine kinase, they point to the role of receptors containing immunoreceptor tyrosine-based activation motifs, which after phosphorylation by SFKs recruit spleen tyrosine kinase for further signal propagation. We propose that this class of receptors triggers the events resulting in increased pro-IL-1β synthesis and disease initiation and/or progression.

Cytokine-driven inflammation is a critical component of the immune system's defense mechanisms. However, its dysregulation can cause a severe harm to the host. To date, a number of mutations compromising the regulation of proinflammatory cytokine production have been identified. In extreme cases, deregulated cytokine secretion caused by these mutations can result in spontaneous inflammation and severe disease. One of the frequently affected cytokines capable of driving pathological inflammation is interleukin-1β (IL-1β). Mutations to the genes controlling the level of its secretion often lead to autoinflammatory disorders characterized by seemingly unprovoked fever attacks and sterile inflammatory damage to various organs and tissues (1). While it is clear that deregulated production of IL-1β is key part of this pathological response, the initiating events that trigger harmful inflammation remain unknown in many cases. They may include excessive response to innocuous endogenous ligands or microbiota or receptor-independent activity of proinflammatory signaling pathways.

In order to understand the mechanisms of autoinflammatory diseases, mouse models proved highly valuable. One of the best studied is the mouse strain known as *Pstpip2*^*cmo*^ (2). This strain harbors a point mutation in *Pstpip2* gene resulting in complete absence of corresponding PSTPIP2 protein (3, 4). Its deficiency leads to chronic multifocal osteomyelitis (CMO), an autoinflammatory disease characterized by inflammatory bone damage and soft tissue swelling localized mainly to hind paws and tail area. The disease resembles several human autoinflammatory disorders, including chronic recurrent multifocal osteomyelitis and synovitis–acne–pustulosis–hyperostosis–osteitis syndrome (2, 5). CMO development in *Pstpip2*^*cmo*^ mouse strain can be prevented by inactivation of the genes coding for IL-1β or its receptor (6, 7, 8), demonstrating that IL-1β is critical for disease initiation. The disease can also be prevented by inactivation of *Myd88* gene essential for signal transduction by IL-1 receptor (9). In addition, increased production of active IL-1β was observed in affected tissues and in *Pstpip2*^*cmo*^ neutrophils (6, 7, 8, 10), a cell type critical for triggering this disease (7, 9). These results clearly demonstrated that IL-1β is a crucial element of the mechanism driving spontaneous inflammation and bone damage in *Pstpip2*^*cmo*^ mouse strain. However, similar to a number of other autoinflammatory diseases, the initial triggering event remains unclear. Gut microbiota may play a role, since it has been shown that their altered composition is important for the disease development in *Pstpip2*^*cmo*^ mice (7). In addition, excessive reactive oxygen species (ROS) production by *Pstpip2*^*cmo*^ neutrophils has been observed *in vivo* in tissues typically affected by the disease weeks before the first visible symptoms, suggesting that *Pstpip2*^*cmo*^ neutrophils are responding to a so far unknown stimulus in the affected tissues early on during the disease development (9).

Since PSTPIP2 is an adaptor protein, its function is likely dependent on its interaction partners. These include all members of the PEST family of protein tyrosine phosphatases, phosphoinositide phosphatase SHIP1, and inhibitory kinase Csk, key negative regulator of Src-family kinases (SFKs) (10, 11). To what extent each partner contributes to IL-1β regulation and CMO development has not been studied so far. However, these interactions suggest that PSTPIP2 regulates signaling pathways dependent on protein tyrosine (and phosphoinositide) phosphorylation. Csk and PEST-family PTPs were shown to bind each other and cooperate in the negative regulation of SFK (12, 13, 14, 15). SFKs are critical for initiation of signaling by a number of key leukocyte receptors, including those expressed by neutrophils (16). Moreover, there is a growing evidence about regulation of a major activator of IL-1β processing, NLRP3 inflammasome, by SFKs and downstream protein tyrosine kinases (17, 18, 19, 20, 21, 22, 23).

Crossbreeding of *Pstpip2*^*cmo*^ mice with strains lacking components of IL-1β pathway proved a useful strategy in determining the roles of these molecules in disease development (6, 7, 8, 24, 25). However, it is not possible to use this strategy for SFK. Neutrophils express almost all Src family members, which are to a significant extent redundant with each other (26). It is not technically feasible to genetically inactivate all these kinases simultaneously. Therefore, to analyze the role of SFKs in CMO disease outcome, we decided to crossbreed *Pstpip2*^*cmo*^ mice with mice lacking receptor-like protein tyrosine phosphatase CD45 encoded by *Ptprc* gene. CD45 is an abundantly expressed surface glycoprotein in the cells of hematopoietic origin. One of the major roles of CD45 phosphatase is in allowing the activation of SFK by dephosphorylation of their C-terminal inhibitory tyrosine (27). It counterbalances the effect of Csk kinase, which is the main negative regulator of SFKs and binding partner of PSTPIP2 (10, 28). By inactivating *Ptprc* gene in *Pstpip2*^*cmo*^ mice, we aimed at reducing SFK activity in leukocytes of these mice by increasing the phosphorylation of their inhibitory tyrosines to obtain evidence supporting the involvement of SFK-dependent signaling in CMO development. We show that while deficiencies in components of Toll-like receptor signaling pathways do not affect CMO development, deficiency in CD45 phosphatase lowers IL-1β levels in *Pstpip2*^*cmo*^ mice leading to mitigation of osteomyelitis and tissue inflammation. These data suggest an important role of CD45 phosphatase in the regulation of the signaling pathways leading to enhanced IL-1β production and imply SFK-dependent receptors in the development of autoinflammatory osteomyelitis. At the same time, they bring evidence against the major role of TLRs.

## Results

### MYD88- and TRIF-mediated signaling are dispensable for CMO development while CD45 plays an important role

**Figure 1:**
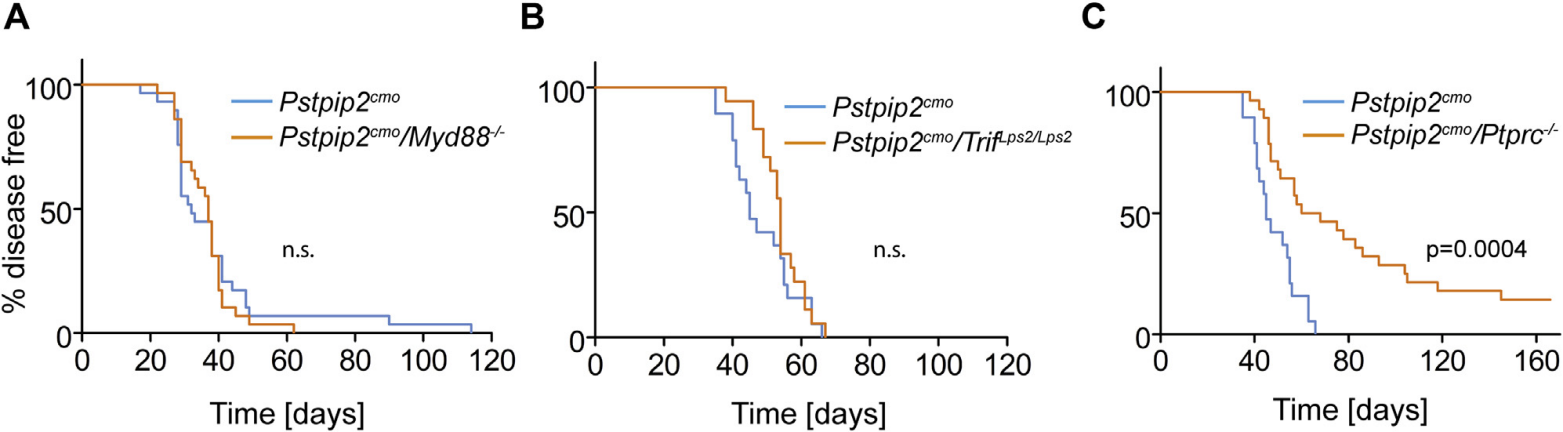
**CD45 deficiency but not deficiencies in Toll-like receptor signaling adaptors attenuate autoinflammatory osteomyelitis progression in *Pstpip2***^***cmo***^**mice.***A*, WT mice were lethally irradiated and transplanted with bone marrow from *Pstpip2*^*cmo*^ or *Pstpip2*^*cmo*^*/Myd88*^*−/−*^ mice. Appearance of diseases symptoms was followed for 120 days (n = 29). *B* and *C*, time of disease symptom appearance in *Pstpip2*^*cmo*^, *Pstpip2*^*cmo*^*/Trif*^*-Lps2/Lps2*^, and *Pstpip2*^*cmo*^*/Ptprc*^*−/−*^ mice (n > 18). In (*B*) *Pstpip2*^*cmo*^ and *Pstpip2*^*cmo*^*/Trif*^*-Lps2/Lps2*^ mice are compared, whereas in (*C*), the same *Pstpip2*^*cmo*^ mice are compared with *Pstpip2*^*cmo*^*/Ptprc*^*−/−*^.

First, we compared the effects of MyD88-/TRIF-dependent Toll-like receptor signaling and signaling dependent mainly on protein tyrosine phosphorylation in the development of autoinflammatory osteomyelitis. To do this, we crossbred *Pstpip2*^*cmo*^ mouse strain with strains deficient in key components of these pathways, including adaptor proteins MyD88 (29) and TRIF (30) and receptor protein tyrosine phosphatase CD45 (encoded by *Ptprc* gene) (31). As a result, we obtained three double-mutant strains *Pstpip2*^*cmo*^*/Myd88*^*−/−*^, *Pstpip2*^*cmo*^*/Trif*^*Lps2/Lps2*^, *and Pstpip2*^*cmo*^*/Ptprc*^*−/−*^. We have shown previously that *Pstpip2*^*cmo*^*/Myd88*^*−/−*^ mice do not develop any symptoms of the disease, demonstrating the key role of this adapter in CMO development (9). It can be likely explained by its involvement in IL-1 receptor signaling, which is required for the disease development in *Pstpip2*^*cmo*^ mice. Importantly, expression of IL-1 receptor on nonhematopoietic (radioresistant) cells is required while its expression on radiosensitive hematopoietic cells does not appear to play a role in CMO (24). This allowed us to analyze the contribution of leukocyte-expressed TLR/MyD88 to the disease development using bone marrow chimeras. We performed bone marrow transplantation from young asymptomatic *Pstpip2*^*cmo*^ or *Pstpip2*^*cmo*^/*Myd88*^*−/−*^ donors into lethally irradiated WT recipients. Unexpectedly, we observed complete disease development without any delay in disease progression, regardless of the donor cell origin (Fig. 1*A*). Since TRIF is not critical for IL-1 receptor signaling, we could analyze the role of TRIF directly in *Pstpip2*^*cmo*^*/Trif*^*Lps2/Lps2*^ mice without the transplantation. *Pstpip2*^*cmo*^*/Trif*^*Lps2/Lps2*^ mice developed the CMO disease with identical kinetics as *Pstpip2*^*cmo*^ mice (Fig. 1*B*). These data suggest that priming of leukocytes through TLR/MyD88 or TLR/TRIF signaling does not play any major role in CMO development since *Pstpip2*^*cmo*^ hematopoietic cells without functional MyD88 or TRIF adaptors were fully capable of driving the autoinflammation. This result also confirmed the earlier finding that IL-1 receptor on hematopoietic cells is dispensable for CMO development (24). In contrast, monitoring of *Pstpip2*^*cmo*^*/Ptprc*^*−/−*^ mice revealed that the disease development in these mice was significantly delayed with part of the mice remaining healthy throughout the experiment (Fig. 1*C*). These results suggested that protein tyrosine phosphorylation regulated by CD45 contributes to CMO development.

### Symptoms of autoinflammation in *Pstpip2*^*cmo*^ mice are milder in the absence of CD45

**Figure 2:**
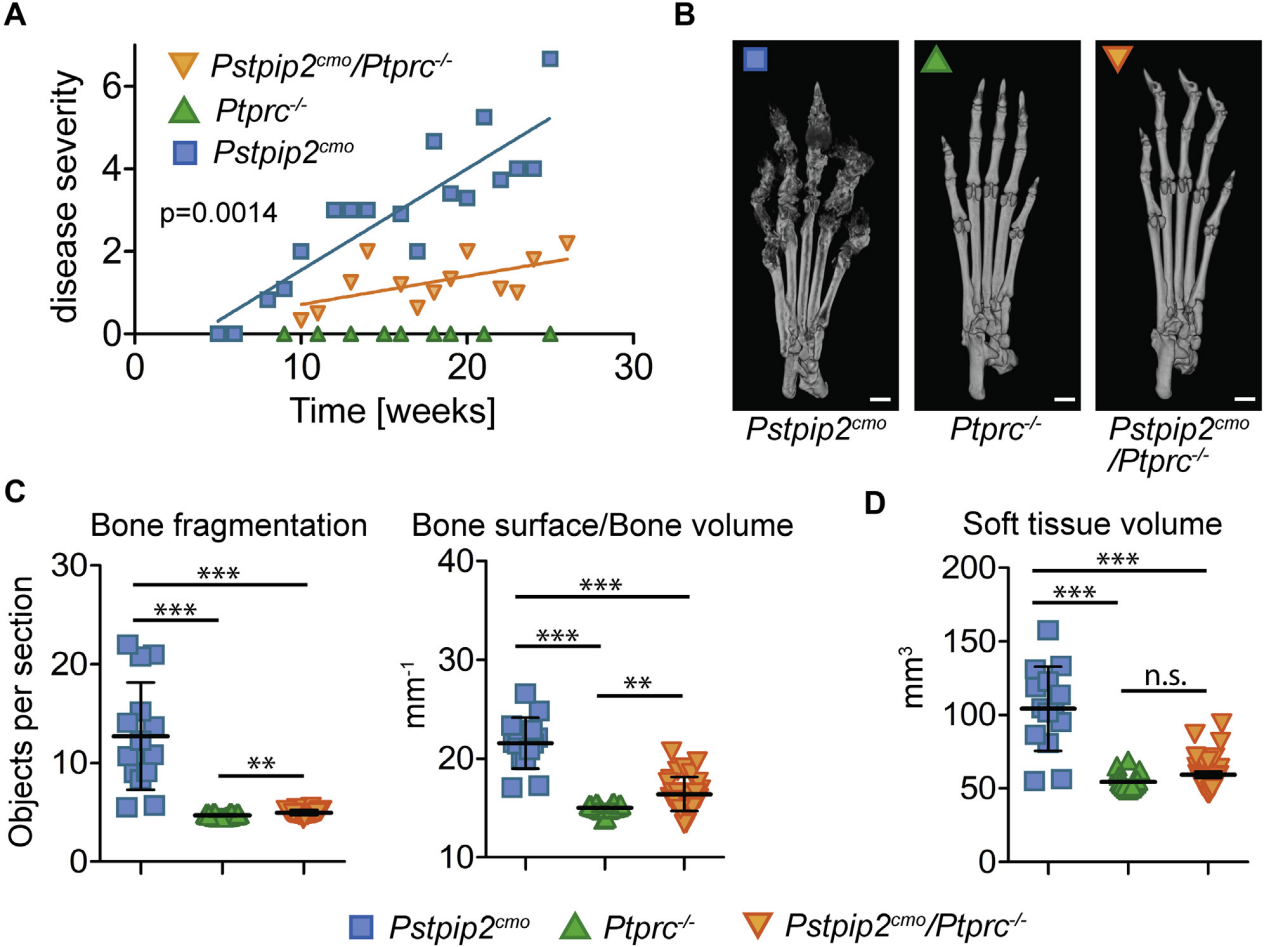
**Milder disease symptoms in CD45-deficient *Pstpip2***^***cmo***^**mice.***A*, disease severity scored by visual inspection of the hind paw photographs collected over the course of this study (scale from 0 to 8). Each point is a mean value representing the mice of the same age and genotype. Lines were generated using linear regression. *B*, representative X-ray μCT scans of hind paw bones from 20-week-old mice. The scale bar represents 1 mm. *C*, quantification of bone damage from X-ray μCT scans obtained from multiple mice. Two different parameters were calculated, bone fragmentation and the ratio of bone surface and volume. *D*, volume of soft tissue in hind paws calculated from X-ray μCT data. Values were obtained by subtracting bone volume from a total paw volume (total paw volume is the volume of the paw reconstructed from μCT scans, including all soft tissues and the bone).

To better understand the effects of CD45, we performed more careful analysis of *Pstpip2*^*cmo*^*/Ptprc*^*−/−*^ mice. Symptom evaluation revealed that the severity of the disease was substantially milder than in *Pstpip2*^*cmo*^ strain (Fig. 2*A*). Microcomputerized tomography (μCT) scans of hind paws demonstrated that bone damage was significantly lower than in *Pstpip2*^*cmo*^ mice, though still present (Fig. 2, *B* and *C*). Soft tissue swelling was also detected by μCT in some animals, although for the entire group as the whole it did not reach the level of statistical significance because of the presence of a number of animals where the swelling was only mild or not observed at all (Fig. 2*D*). These data demonstrate that CMO disease is clearly present in CD45-deficient *Pstpip2*^*cmo*^ mice. However, its severity is significantly lower than in *Pstpip2*^*cmo*^ mice expressing CD45.

### Phosphorylation of SFK inhibitory tyrosine is increased in the absence of CD45, while the loss of PSTPIP2 does not have any effect

**Figure 3:**
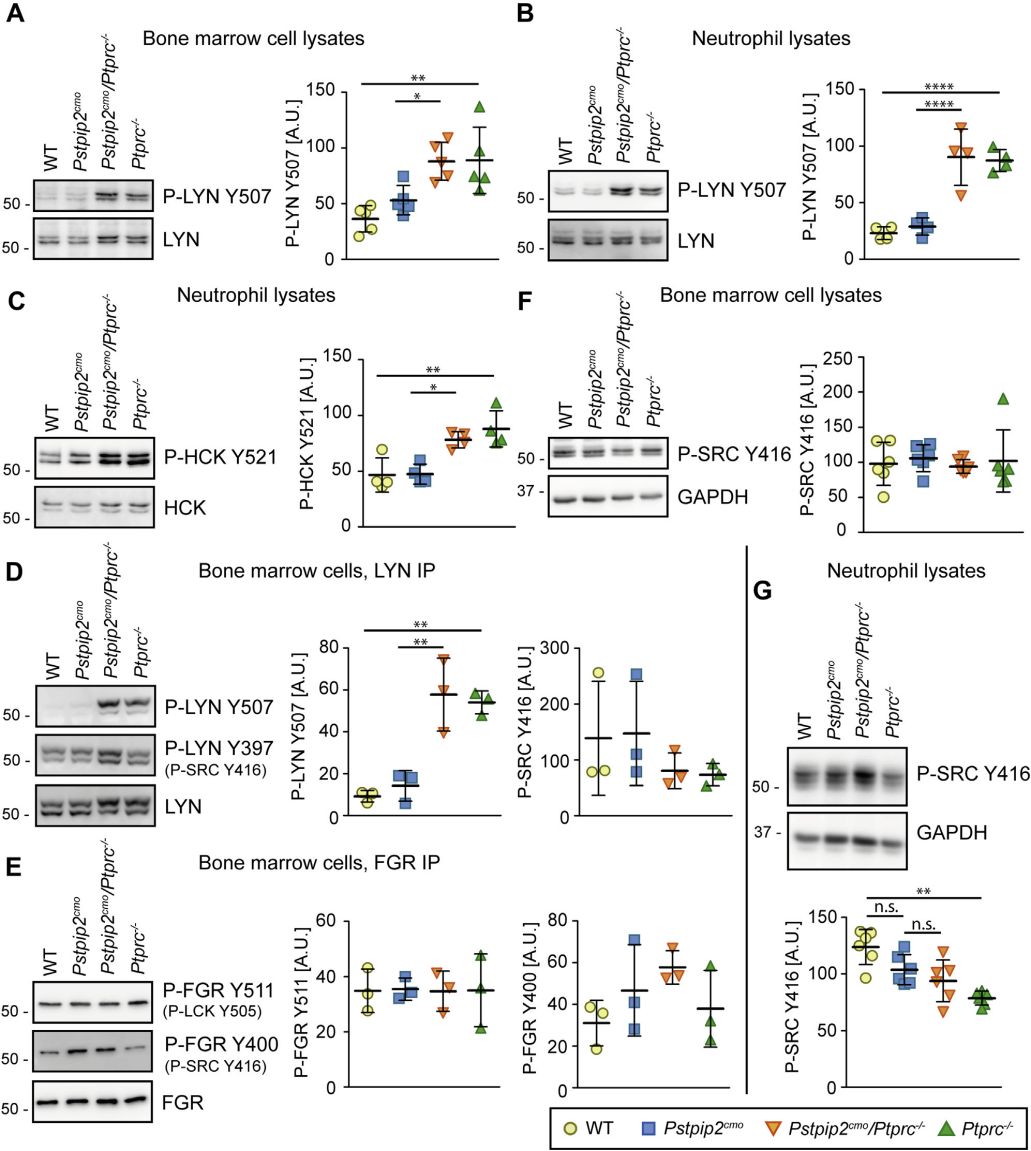
**Inhibitory tyrosine of Src-family kinases LYN and HCK is hyperphosphorylated in *Ptprc***^***−/−***^**and *Pstpip2***^***cmo***^***/Ptprc***^***−/−***^**cells.***A*, lysates of bone marrow cells from mice of indicated genotypes were subjected to immunoblotting with antibody to inhibitory phosphotyrosine of LYN (P-LYN Y507). *B*, similar experiment as in (*A*) on purified neutrophils. *C*, lysates of purified neutrophils from mice of indicated genotypes were subjected to immunoblotting with antibody to inhibitory phosphotyrosine of HCK (P-HCK Y521). *D* and *E*, LYN (*D*) or FGR (*E*) was immunoprecipitated from the lysates of bone marrow cells obtained from mice of indicated genotypes, followed by immunoblotting with antibodies to their inhibitory and activating phosphotyrosines. In case of FGR, inhibitory phosphorylation was detected with antibody to similar phosphotyrosine of LCK (P-LCK Y505). For detection of activating phosphorylation, antibody to P-SRC Y416 crossreacting with multiple Src-family members was used in both (*D*) and (*E*). *F*, lysates of bone marrow cells from mice of indicated genotypes were subjected to immunoblotting with antibody to activating phosphotyrosine of multiple SFK (P-SRC Y416). *G*, similar experiment as in (*F*) on purified neutrophils. For each experiment, representative immunoblot and quantification of multiple experiments is shown. *A*–*E*, phospho-SFK signals were normalized to total SFK. *F* and *G*, because of the crossreactivity of P-SRC Y416 antibody with multiple Src family members, the signal was normalized to GAPDH. To allow better comparison of multiple experiments in (*F*) the obtained values were further normalized to experiment average. A.U., arbitrary units; IP, immunoprecipitation.

CD45 is a major activator of SFKs, since it dephosphorylates their C-terminal inhibitory tyrosine (27). In addition, PSTPIP2 *via* binding to Csk, a kinase phosphorylating this tyrosine, is expected to inhibit SFK activity (10, 32, 33). To assess the effects of the loss of CD45 and PSTPIP2 on SFK phosphorylation at their inhibitory tyrosines, we prepared lysates from bone marrow cells and purified neutrophils of mice carrying these mutations and probed for phosphorylation of these sites. We focused on the three most important myeloid cell SFKs LYN, HCK, and FGR. As expected, in both, bone marrow cells and purified neutrophils, CD45 deficiency resulted in a substantial increase in phosphorylation detected by antibody to inhibitory tyrosine of SFK LYN (Fig. 3, *A* and *B*). For HCK inhibitory tyrosine, we only obtained reliable signal from purified neutrophils. There, the pattern of phosphorylation was similar to Lyn (Fig. 3*C*). To further verify these results, we immunoprecipitated LYN from bone marrow cell lysates followed by staining with antibody to LYN inhibitory tyrosine. Similar to the whole cell lysates, LYN was hyperphosphorylated on inhibitory tyrosine in both samples lacking CD45 (Fig. 3*D*). Unfortunately, three different antibodies to HCK we tested did not immunoprecipitate murine HCK, and so for HCK, we could not perform this experiment. To our knowledge, there are no reliable phosphospecific antibodies to Fgr inhibitory tyrosine. However, we found that after immunoprecipitation with FGR-specific antibody, it is recognized by antibody to inhibitory tyrosine of SFK LCK, C terminus of which shows sequence homology to FGR. Surprisingly, CD45 deficiency did not have any effect on FGR phosphorylation detected by this antibody (Fig. 3*E*). The absence of CD45 only mildly affected phosphorylation of the SFK activating tyrosine (Fig. 3, *D*–*G*). Interestingly, the presence or the absence of PSTPIP2 did not alter phosphorylation of any of these SFK (Fig. 3, *A*–*G*).

### CD45 deficiency does not affect generation of ROS

**Figure 4:**
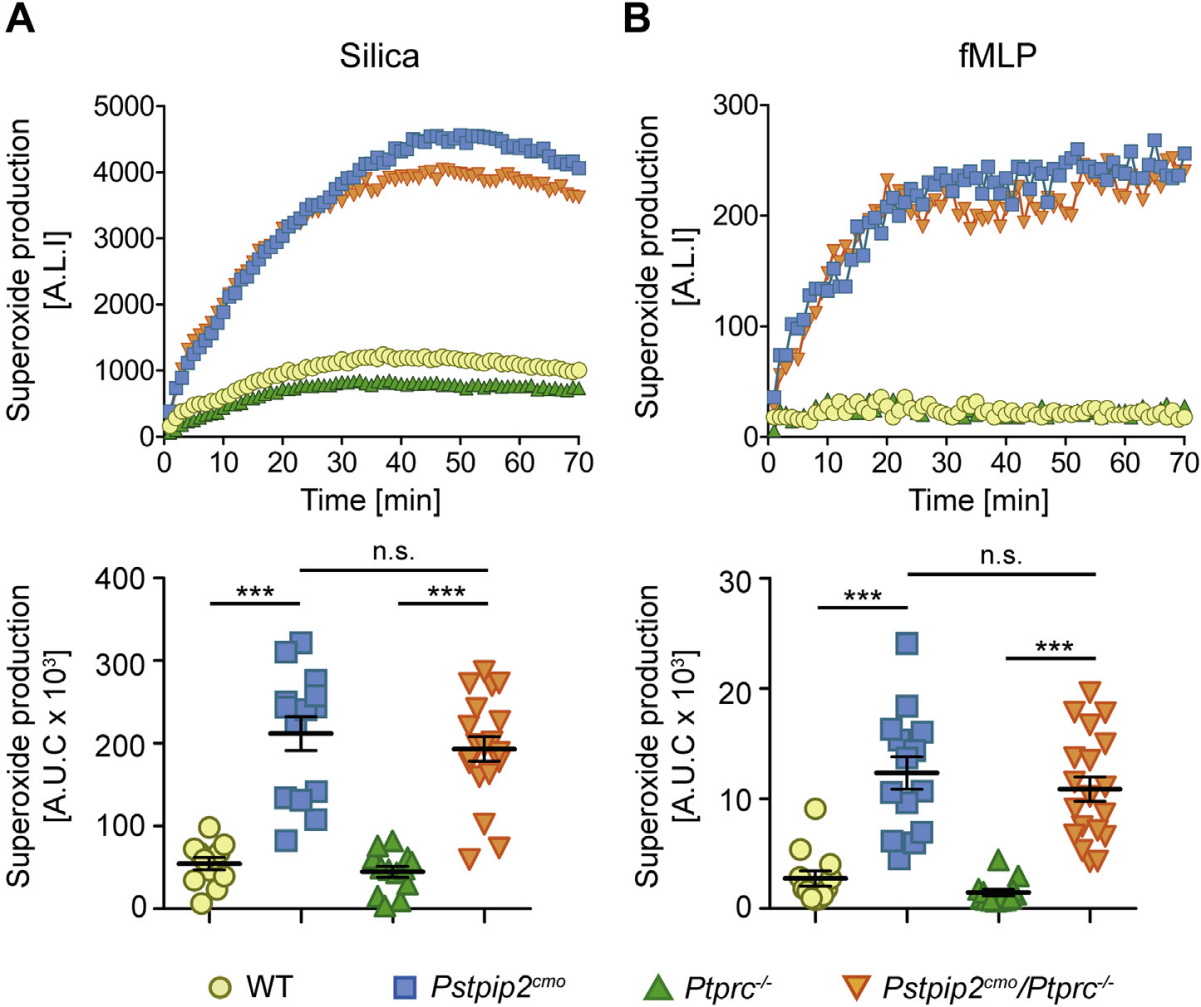
**Similar dysregulation of ROS production in *Pstpip2***^***cmo***^**and *Pstpip2***^***cmo***^***/Ptprc***^***−/−***^**mice.***A* and *B*, representative time course and area under the curve quantification of multiple time-course measurements of superoxide production by bone marrow cells from mice of indicated genotypes. The cells were activated by silica particles in (*A*) and by fMLP in (*B*). A.L.I., arbitrary luminescence intensity; A.U.C., area under the curve.

So far, two important processes dysregulated in *Pstpip2*^*cmo*^ mice have been shown to contribute to CMO disease severity. These are generation of ROS by phagocyte NADPH oxidase and production of IL-1β mediated by NLRP3 inflammasome, caspase-8, and neutrophil proteases (6, 7, 8, 9, 24, 25). Our measurements of ROS production in bone marrow cells after silica or fMLP exposure confirmed previous observations of substantially increased superoxide levels generated by *Pstpip2*^*cmo*^ cells. Surprisingly, superoxide production by *Pstpip2*^*cmo*^*/Ptprc*^*−/−*^ cells was increased to a similar extent, and no significant difference between *Pstpip2*^*cmo*^ and *Pstpip2*^*cmo*^*/Ptprc*^*−/−*^ was detected (Fig. 4, *A* and *B*).

### Reduced IL-1β production in CD45-deficient mice

**Figure 5:**
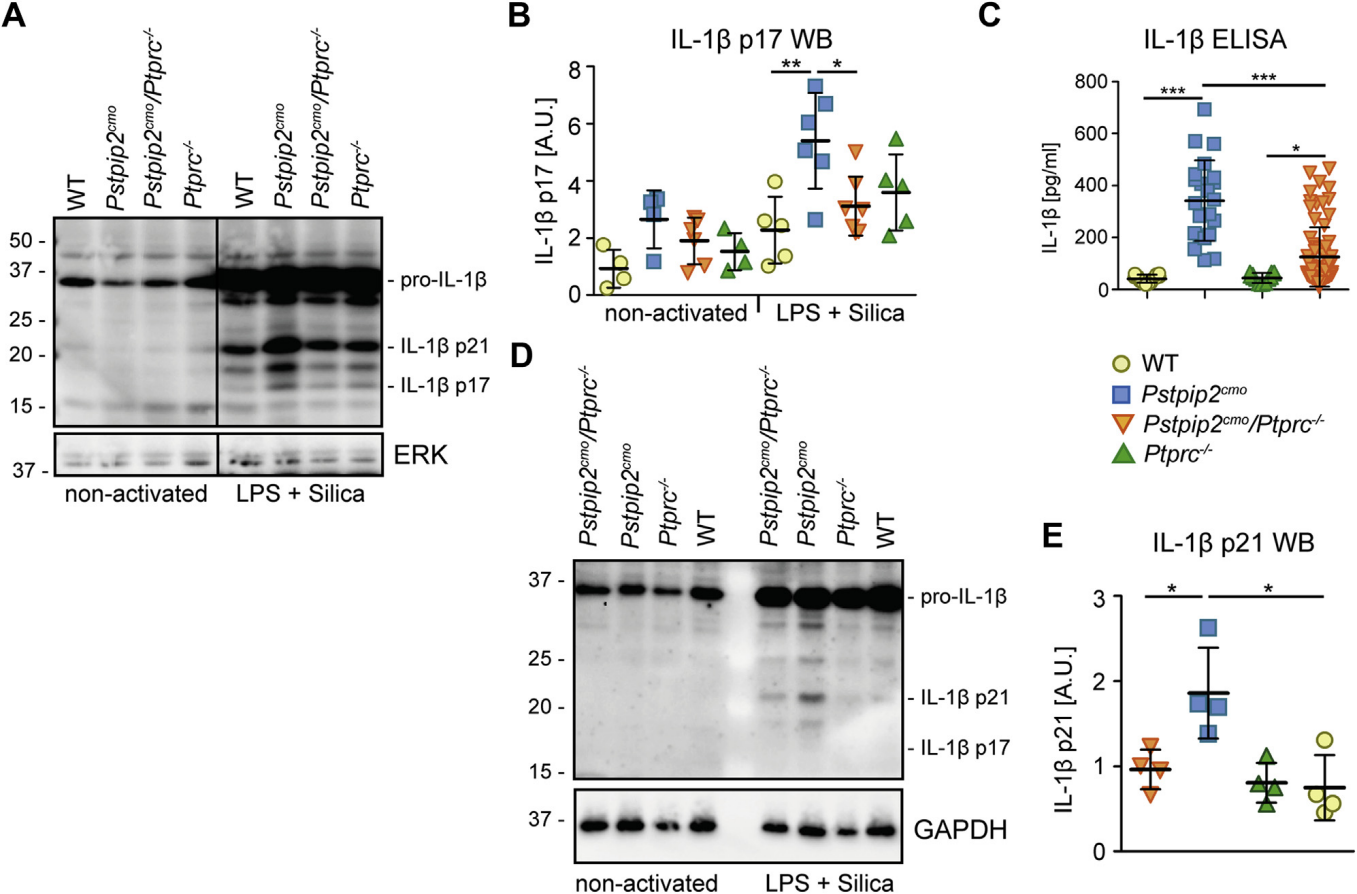
**CD45 deficiency results in a significant reduction of active IL-1β production in *Pstpip2***^***cmo***^**mice.***A* and *B*, IL-1β processing to active IL-1β p17 was analyzed by immunoblotting of bone marrow cells activated by LPS and silica. Representative Western blot (*A*) and quantification of multiple experiments (normalized to ERK and reference sample loaded on each gel) (*B*) are shown. *C,* IL-1β in the footpad homogenates from 20-week-old mice of indicated genotypes was quantified by ELISA. *D* and *E*, IL-1β processing in purified neutrophils activated by LPS and silica was analyzed by immunoblotting of the whole cell lysates. Representative Western blot (*D*) and quantification of p21 signal from multiple experiments normalized to GAPDH signal (*E*) are shown. Vertical line in (*A*) separates samples that were at different positions on the same immunoblot membrane. A.U., arbitrary units.

In contrast to the ROS production, exacerbated production of processed IL-1β p17 observed after activation of inflammasome by silica particles in *Pstpip2*^*cmo*^ bone marrow cells was significantly reduced in *Pstpip2*^*cmo*^*/Ptprc*^*−/−*^ cells (Fig. 5, *A* and *B*). In addition, we also observed substantially reduced IL-1β levels *in vivo* in the footpads of *Pstpip2*^*cmo*^*/Ptprc*^*−/−*^ mice when compared with *Pstpip2*^*cmo*^ (Fig. 5*C*). Neutrophils are the most critical cell type indispensable for disease development in *Pstpip2*^*cmo*^ mice (7, 9). However, we did not observe any p17 in neutrophil lysates after induction of pro-IL-1β production by lipopolysaccharide (LPS) followed by inflammasome activation by silica (Fig. 5*D*). Nor could we detect any inflammasome-generated caspase-1 p20 or any significant differences in gasdermin D cleavage between *Pstpip2*^*cmo*^ and *Pstpip2*^*cmo*^*/Ptprc*^*−/−*^ cells that could help explain differences in disease severity between these two strains (Fig. S1). On the other hand, we could observe IL-1β p21 thought to be generated *via* cleavage of pro-IL-1β by neutrophil proteases (34) with the highest levels in *Pstpip2*^*cmo*^ neutrophils that were significantly reduced in *Pstpip2*^*cmo*^*/Ptprc*^*−/−*^ cells (Fig. 5, *D* and *E*).

**Figure 6:**
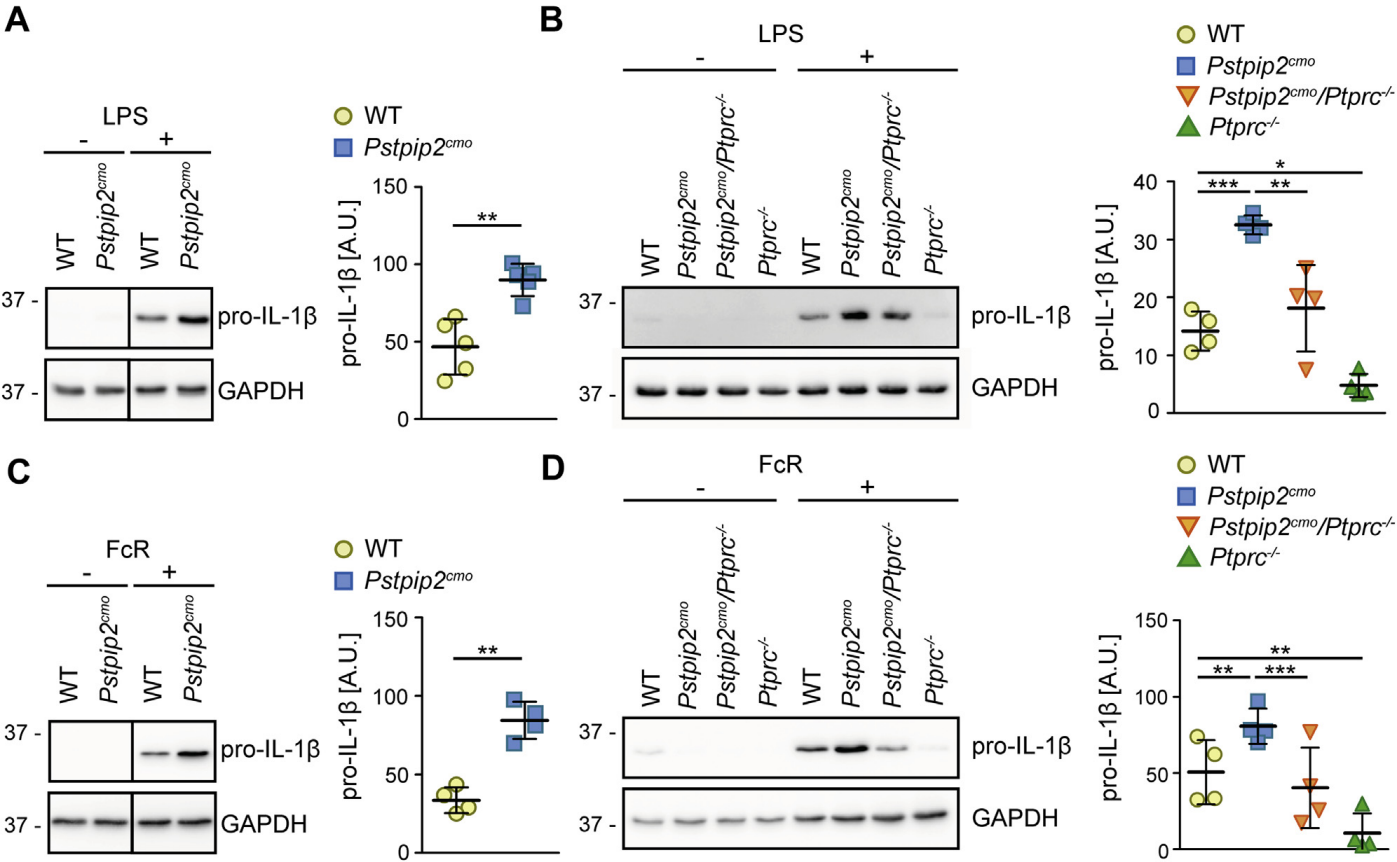
**Enhanced production of pro-IL-1β in *Pstpip2***^***cmo***^**mice and its attenuation by CD45 deficiency.***A*, lysates of bone marrow cells from WT and *Pstpip2*^*cmo*^ mice activated with a low dose of LPS (10 ng/ml) were subjected to immunoblotting with antibody to pro-IL-1β. *B*, similar experiment as in (*A*) on lysates of purified neutrophils from mice of indicated genotypes. *C*, lysates of Fc receptor–activated bone marrow cells from WT and *Pstpip2*^*cmo*^ mice were subjected to immunoblotting with antibody to pro-IL-1β. *D*, similar experiment as in (*C*) on lysates of purified neutrophils from mice of indicated genotypes. For each experiment, representative immunoblot and quantification of multiple experiments after normalization to GAPDH signal are shown. *Vertical line* in (*A*) and (*C*) separates samples that were at different positions on the same immunoblot membrane. A.U., arbitrary units.

Published observations demonstrating the roles of multiple caspases and neutrophil proteases in CMO disease development (7, 24) suggested that there might be a step upstream of all these factors that is dysregulated in *Pstpip2*^*cmo*^ mice. This notion prompted us to investigate the production of IL-1β precursor—pro-IL-1β. Our previous observations suggested that LPS-induced pro-IL-1β production is not altered in *Pstpip2*^*cmo*^ bone marrow cells and neutrophils (10). However, relatively high LPS doses were used in those experiments. When we used lower dose of 10 ng/ml LPS, we detected a significantly higher pro-IL-1β production in *Pstpip2*^*cmo*^ bone marrow cells when compared with WT cells (Fig. 6*A*). In addition, we also detected deregulated LPS-induced production of pro-IL-1β in purified *Pstpip2*^*cmo*^ neutrophils (Fig. 6*B*). Importantly, CD45 deficiency significantly attenuated this production. Given the dependence of LPS-triggered signaling on MYD88, our data in Figure 1*A* make LPS an unlikely candidate for CMO triggering factor in *Pstpip2*^*cmo*^ mice *in vivo*. On the other hand, published data on the critical importance of spleen tyrosine kinase (SYK) (35), as well as the data presented in this article, suggest an involvement of an immunoreceptor tyrosine-based activation motif (ITAM)-containing receptor. As a model of these receptors we selected Fc receptors, where the signaling is dependent on the ITAM motif in the receptor gamma chain. First, we tested if Fc receptor crosslinking results in any production of pro-IL-1β in bone marrow cells. This experiment demonstrated that Fc receptor activation is capable of triggering pro-IL-1β synthesis in these cells. Importantly, its production was significantly higher in bone marrow cells from *Pstpip2*^*cmo*^ mice (Fig. 6*C*). Next, we performed a similar experiment on purified neutrophils from mice of all four genotypes. This experiment again showed enhanced production of pro-IL-1β by *Pstpip2*^*cmo*^ neutrophils, which was significantly attenuated in CD45-deficient *Pstpip2*^*cmo*^*/Ptprc*^*−/−*^ cells (Fig. 6*D*). This experiment suggested an involvement of ITAM-containing receptors in disease initiation in *Pstpip2*^*cmo*^ mice and a role of pro-IL-1β generation in this process.

## Discussion

In mice, PSTPIP2 deficiency results in CMO, an autoinflammatory disease driven by deregulated IL-1β and ROS production by neutrophils (6, 7, 8, 9, 24, 25). The signaling event that triggers the disease onset *in vivo* is currently unknown. Because of the strong effect of microbiota on the disease course (7), we have suspected that one or more TLRs might be involved in its initiation. However, our analysis of *Pstpip2*^*cmo*^ mice with deficiencies in essential TLR signaling adaptors MYD88 in hematopoietic cells or TRIF in the whole body demonstrated that the disease development is triggered with unchanged kinetics even when they are inactivated. It should be noted that there may be a certain level of redundancy between MYD88 and TRIF, and so their role still cannot be completely excluded. On the other hand, the loss of CD45, which results in downregulation of the activity of SFK-dependent pathways, resulted in delayed kinetics and alleviation of the disease symptoms. Apart from SFK, CD45 has been reported to dephosphorylate other substrates, including TCRζ, SKAP55, DAP12, JAK kinases, and PAG/Cbp (36, 37, 38, 39). However, to our knowledge, SFKs are the only enzymes where CD45 has positive regulatory function (either direct or *via* some of the aforementioned substrates). Reduced disease severity observed in *Pstpip2*^*cmo*^*/Ptprc*^*−/−*^ mice is consistent with the loss of activating effect of CD45, rather than lack of inhibitory function associated with substrates other than SFK. These observations strongly support the hypothesis that symptom alleviation is caused by reduced activity of SFK. Importantly, Dasari *et al.* (35) recently demonstrated that another protein tyrosine kinase, SYK, is also essential for triggering the disease in *Pstpip2*^*cmo*^ mice. Both SFK and SYK are key components of ITAM signaling pathways (40). Hence, these results suggest that the exaggerated signaling leading to CMO disease may be initiated by an ITAM-containing receptor. There are number of these receptors in neutrophils, including Fc receptors, dectins, integrins, paired immunoglobulin-like receptors/leukocyte immunoglobin-like receptors, TARM1, TREM-1/2, and other FcRγ chain or DAP12-associated receptors, number of which have endogenous ligands (16, 41, 42, 43, 44). We have shown previously that Fc receptor signaling is deregulated in *Pstpip2*^*cmo*^ mice. However, it is possible that other ITAM-dependent receptors could be similarly affected by PSTPIP2 deficiency, since they share the same basic signaling mechanisms.

Interestingly, we have not observed any changes in SFK phosphorylation in *Pstpip2*^*cmo*^ mice. These results favor the interpretation that the dysregulation of signaling caused by the absence of PSTPIP2 is not at the level of SFK activity or ITAM phosphorylation by SFK but rather further downstream in this pathway. In such case, ITAM-containing receptors themselves still could be essential for disease development, yet not directly deregulated in the absence of PSTPIP2. This interpretation would also be consistent with a rather generalized hypersensitivity of *Pstpip2*^*cmo*^ neutrophils to a broad range of different stimuli observed previously (9, 10). However, precisely which part of the ITAM-dependent signaling cascade is affected by PSTPIP2 deficiency remains still unclear.

We have observed increased production of active IL-1β p17, typically generated by inflammasome, in nonseparated *Pstpip2*^*cmo*^ bone marrow cells. On the other hand, in purified neutrophils, we were unable to detect this protein. Nevertheless, neutrophils were shown to be absolutely required for CMO disease development in *Pstpip2*^*cmo*^ mice (7, 9). It is possible that deregulated activity of *Pstpip2*^*cmo*^ neutrophils promotes production of active IL-1β by monocytes present in the same bone marrow cell samples or that neutrophil's own production is below Western blot detection limit but still present and compensated for by large neutrophil numbers *in vivo*. There are multiple pathways of pro-IL-1β processing and production of active protein involved in CMO development in mice, including NLRP3 inflammasome/caspase-1, additional mechanism involving caspase-8, and neutrophil proteases (multiple have been tested in *Pstpip2*^*cmo*^ mice, including elastase, proteinase 3, cathepsins B, C, G) (7, 24). Genes for all these proteins have been individually inactivated in *Pstpip2*^*cmo*^ mice without any effect on disease development (with the exception of limited but significant disease alleviation in case of cathepsin C, which is known to be an essential upstream activator of the other neutrophil proteases) (7, 8, 24, 45). Importantly, combined deficiency of the individual inflammasome components and caspase-8 almost completely prevented disease development (7, 24). These data suggested that any of the two pathways, (*i.e.*, NLRP3 inflammasome or caspase-8) can drive the disease on its own with some contribution from neutrophil proteases, whereas none of these pathways alone is critical because of their mutual redundancy. There may be more efficient activators of these pathways in neutrophils than silica. Silica has been frequently used as a model inflammasome activator in studies of *Pstpip2*^*cmo*^ mice (7, 8, 9, 10), but it is unlikely to be responsible for the CMO disease initiation *in vivo*. There are many other inflammasome activators of both endogenous and exogenous origin (46), some of which may be more potent activators in neutrophils and also more important *in vivo*. Inflammasome activation triggered by these activators does not have to be dysregulated in *Pstpip2*^*cmo*^ neutrophils for the disease to develop. The dysregulation at the level of synthesis of pro-IL-1β, a precursor of active IL-1β, would likely be sufficient to drive the disease development in *Pstpip2*^*cmo*^ mice. Dysregulation at this upstream step could explain the redundancy of multiple downstream pathways of pro-IL-1β processing into active protein.

Increased pro-IL-1β production by *Pstpip2*^*cmo*^ neutrophils can be triggered not only by LPS exposure but also by Fc receptor stimulation. Consistent with ITAM/SFK role in the disease development discussed previously, it is likely that Fc or other ITAM-dependent receptor is involved in disease initiation *in vivo*. Fc receptor signaling pathway is well defined, and CD45 and SFK are both important players in Fc receptor signaling in myeloid cells (28, 40, 47). Thus, our current knowledge leads to a conclusion that the reduced SFK activity in CD45-deficient *Pstpip2*^*cmo*^ cells results in attenuated Fc receptor signaling and diminished Fc receptor-dependent pro-IL-1β production. Given the universality of the basic principles of ITAM-mediated signaling, similar mechanism, potentially involving other ITAM-bearing receptors, is also likely at play *in vivo*.

In summary, based on our findings and previously published data, we propose a hypothesis where PSTPIP2 negatively regulates a step common to multiple signaling pathways in neutrophils, among which ITAM-dependent signaling plays a key role. A so far unknown endogenous ligand/ligands or, perhaps, even tonic signals in the absence of any ligand trigger ITAM-dependent signaling, which after reaching the step regulated by PSTPIP2, becomes exacerbated and drives increased pro-IL-1β production making more of this precursor available for inflammasome, caspase-8, and neutrophil protease cleavage. Increased IL-1β generation then leads to disease development. In the absence of CD45, SFKs become hyperphosphorylated on their inhibitory tyrosines, which results in their reduced activity, reduced ITAM signaling, and pro-IL-1β production, ultimately resulting in disease alleviation. The identity of the pathway step directly regulated by PSTPIP2 still remains unclear. Available data nevertheless suggest that inhibition of ITAM-mediated signaling by pharmacological inhibitors should be considered as therapeutic approach in similar diseases in humans.

## Experimental procedures

### Antibodies

Rabbit polyclonal antibodies to phospho-LYN Y507 (#2731), phospho-LCK Y505 (#2751), and phospho-Src Family Y416 (#2101), and rabbit monoclonal antibodies to IL-1β (clone D3H1Z; #12507) and gasdermin D (clone E9S1X; #39754) were from Cell Signaling Technology; rabbit polyclonal antibody to phospho-HCK Y521 (PA5-37592) was from Invitrogen, Thermo Fisher Scientific; rabbit polyclonal antibody to GAPDH (G9545) and mouse monoclonal antibody to β-actin (clone AC-74) were from Sigma–Aldrich; rabbit polyclonal antibody to ERK (C-14, sc-154), and mouse monoclonal antibodies to HCK (clone 3D12E10) and FGR (D-6) were from Santa Cruz Biotechnology; mouse monoclonal antibody to caspase-1 (p20, Casper-1) was from AdipoGen Life Sciences. Mouse monoclonal antibody to LYN was a kind gift of Petr Draber, Institute of Molecular Genetics of the Czech Academy of Sciences.

### Mice

*Pstpip2*^*cmo*^ mice on C57Bl/6J genetic background carrying the c.293T→C mutation in the *Pstpip2* gene, which results in a loss of PSTPIP2 protein, were described earlier (9). They were generated from C.Cg-*Pstpip2*^*cmo*^/J mouse strain on Balb/C genetic background (2, 3) obtained from The Jackson Laboratory, by backcrossing for more than ten generations to C57Bl/6J. All *Pstpip2*^*cmo*^ mice and their derivatives used in this study were on C57Bl/6J background. *Ptprc*^*−/−*^ mouse strain backcrossed to C57Bl/6J (B6;129–*Ptprc*^*tm1Holm*^*/H*), lacking the expression of CD45 because of exon 9 deletion (31) was obtained from European Mouse Mutant Archive (48). *Pstpip2*^*cmo*^*/MyD88*^*−/−*^ mouse strain was described earlier (9) and was generated with the use of B6.129P2(SJL)-*Myd88*^*tm1.1Defr*^/J mouse strain (29) obtained from The Jackson Laboratory. TRIF-deficient mouse strain *Trif*^*Lps2/Lps2*^ (30) was a kind gift from B. Beutler. C57Bl/6J inbred strain was obtained from the animal facility of Institute of Molecular Genetics, Academy of Sciences of the Czech Republic. Experiments in this work that were conducted on animals were approved by the Expert Committee on the Welfare of Experimental Animals of the Institute of Molecular Genetics and by the Academy of Sciences of the Czech Republic and were in agreement with local legal requirements and ethical guidelines.

### Cell activation, ROS, and IL-1β detection

Bone marrow cells were isolated from mice (sacrificed by cervical dislocation) by flushing femurs and tibias, cut at the extremities, with PBS containing 2% fetal bovine serum (FBS). Erythrocytes were removed by lysis in ACK buffer (150 mM NH_4_Cl, 0.1 mM EDTA [disodium salt], 1 mM KHCO_3_). Neutrophils were isolated from bone marrow cells using Neutrophil Isolation Kit (Miltenyi Biotec; #130-097-658) according to the manufacturer's instructions followed by separation on Miltenyi AutoMACS magnetic cell sorter (negative selection). Purity of isolated neutrophils was verified by flow cytometry using CD11b, Ly6C, and Ly6G markers. For measurement of ROS (superoxide) production by luminol-based assay (49, 50), bone marrow cells were plated in a black 96-well plate (SPL Life Sciences) at 10^6^ cells per well in IMDM supplemented with 0.2% FBS and rested for 30 min at 37 °C and 5% CO_2_. Then 100 μM luminol and 50 μg/cm^2^ silica or 100 μM luminol and 1 μg/ml fMLP (all from Sigma–Aldrich) were added, and the luminescence was immediately measured on EnVison plate reader (PerkinElmer) every minute for 70 min. For detection of IL-1β p17 and p21 by immunoblotting, cells were plated in 96-well tissue culture plate at 2 × 10^6^ per well in IMDM containing 0.1% FBS and 100 ng/ml LPS. After 3 h at 37 °C and 5% CO_2_, silica at 50 μg/cm^2^ was added for additional 30 min. Then the cells were lysed by adding equal volume of 2× concentrated SDS-PAGE sample buffer followed by 15-s sonication and subjected to immunoblotting with IL-1β antibodies. For detection of pro-IL-1β, 2 × 10^6^ cells in 700 μl IMDM containing 0.1% FBS and 10 ng/ml LPS were placed in low protein-binding microcentrifuge tubes (Thermo Fisher Scientific) and incubated 3 h at 37 °C and 5% CO_2_. Next, the cells were centrifuged, resuspended in 100 μl IMDM containing 0.1% FBS, and lysed by adding equal volume of 2× concentrated SDS-PAGE sample buffer followed by 15-s sonication and immunoblotting with IL-1β antibodies. For Fc receptor activation, the cells were incubated with 50× diluted culture supernatant from 2.4G2 rat hybridoma (51) (American Type Culture Collection) producing antimouse Fc receptor (CD16/CD32) antibodies (30 min on ice, low protein-binding microcentrifuge tubes). Then, the cells were centrifuged, resuspended in 700 μl IMDM containing 0.1% FBS and 5 μg/ml F(ab′)_2_ Mouse Anti-Rat antibody (Jackson ImmunoResearch), and incubated for 3 h at 37 °C and 5% CO_2_. The lysis and immunoblotting procedures were the same as for the aforementioned LPS activation. For measurement of IL-1β concentrations *in vivo*, footpads from mice sacrificed by cervical dislocation were homogenized using Avans AHM1 Homogenizer (30 s, speed 25) in 1 ml radioimmunoprecipitation lysis buffer (20 mM Tris, pH 7.5, 150 mM NaCl, 1% NP-40, 1% sodium deoxycholate, and 0.1% SDS) supplemented with 5 mM iodoacetamide (Sigma) and 100× diluted Protease Inhibitor Cocktail Set III (Calbiochem, Merck). Insoluble material was removed by centrifugation (20,000*g*, 5 min, 2 °C). Protein concentration in the supernatants was determined using Bradford solution (AppliChem) and adjusted to equal level. Concentrations of IL-1β were then determined by Ready-SET-Go! ELISA kit from eBioscience (Thermo Fisher Scientific) according to the manufacturer's instructions.

### Immunoprecipitation of SFKs

About 8 × 10^7^ bone marrow cells were resuspended in 1 ml lysis buffer (50 mM Tris–HCl, pH 7.5, 150 mM NaCl, 1% *n*-dodecyl β-d-maltoside, 100× diluted Protease Inhibitor Cocktail Set III [Calbiochem, Merck], 1000× diluted Diisopropylfluorophosphate [Sigma, Merck] and 50× diluted PhosStop solution made by dissolving 1 PhosStop pellet [Roche] in 200 μl water) and incubated for 30 min on ice. Next the lysates were centrifuged at 25,000*g* for 10 min. About 2 μg antibody per sample were added followed by 1-h incubation on ice. Next, 30 μl of protein A/G agarose resin (Santa Cruz Biotechnology) was added followed by incubation at 4 to 8 °C with rotation for 90 min (in case of LYN, the antibody was first incubated with protein A/G agarose and then added to the lysates). The resin was washed two times (50 mM Tris–HCl, pH 7.5, 150 mM NaCl, 0.1% *n*-dodecyl β-d-maltoside, 100× diluted Protease Inhibitor Cocktail Set III, and 200× diluted PhosStop), and the proteins were eluted using 2× concentrated SDS-PAGE sample buffer.

### Bone marrow transplantations

In bone marrow transplantation experiments, recipient mice were lethally irradiated with a single dose of 7 Gy. After 6 h, mice were injected with 2 × 10^6^ bone marrow cells from *Pstpip2*^*cmo*^ or *Pstpip2*^*cmo*^/*MyD88*^*−/−*^ mice into tail vein. Mice were monitored for the presence of paw swelling and inflammation twice a week.

### X-ray μCT

Hind paws were scanned *in vivo* in X-ray μCT Skyscan 1176 (Bruker) using the following parameters: voltage: 50 kV, current: 250 μA, filter: 0.5 mm aluminium, voxel size: 8.67 μm, exposure time: 2 s, rotation step: 0.3° for 180° total, object to source distance: 119.271 mm, camera to source distance: 171.987 mm, and time of scanning: 30 min. Virtual sections were reconstructed in NRecon software 1.7.1.0 (Bruker) with the following parameters: smoothing = 3, ring artifact correction = 4, and beam hardening correction = 36%. Intensities of interest for reconstruction were in the range from 0.0045 to 0.0900 attenuation units. Same orientation of virtual sections was achieved with the use of the DataViewer 1.5.4 software (Bruker). μCT data analysis was performed using CT Analyser 1.18.4.0 (Bruker). Scans affected by technical artifacts caused by spontaneous movements of animals were excluded from the analysis. Only distal half of the paws (from the half of the length of the longest metatarsal bone to fingertips) were analyzed. Bone fragmentation (Fig. 2*C*) is represented by the average number of bony objects per section. Total object (*i.e.*, distal paw) volume, total bone volume, and total bone surface were computed to compute bone surface/bone volume ratio (Fig. 2*C*) as the second parameter corresponding to bone fragmentation, and volume of the soft tissue (as total volume minus total bone volume—Fig. 2*D*).

### Statistical analysis

The *p* values were calculated with GraphPad Prism software, version 5.04 (Graphpad Software, Inc), using Gehan–Breslow–Wilcoxon test for Figure 1; Kruskal–Wallis test with Dunn's multiple comparison test for Figures 2, *C* and *D*, 4, *A* and *B*, and 5*C*; repeated-measures ANOVA and Bonferroni's multiple comparison post-test for Figures 3, 5*E*, and 6, *B* and *D*; one-way ANOVA and Bonferroni's multiple comparison post-test for Figure 5*B*; paired *t* test, two-tailed, for Figure 6, *A* and *C*. The asterisks represent *p* values as follows: ∗*p* ≤ 0.05, ∗∗*p* ≤ 0.01, ∗∗∗*p* ≤ 0.001, ∗∗∗∗*p* ≤ 0.0001, n.s.—not significant. Error bars in the figures represent mean ± standard deviation.

## Data availability

Representative experiments are shown in the figures. For any additional information, please contact the corresponding author.

## Supporting information

This article contains supporting information.

## Conflict of interest

The authors declare that they have no conflicts of interest with the contents of this article.
