# Supplementary material for: The receptor-type protein tyrosine phosphatase CD45 promotes onset and severity of IL-1β–mediated autoinflammatory osteomyelitis
Source: J Biol Chem. 2021 Aug 27;297(4):101131. doi: 10.1016/j.jbc.2021.101131 (PMC8455366; doi:10.1016/j.jbc.2021.101131)
Supplement: Figure S1 [file mmc1.pdf]

The receptor-type protein tyrosine phosphatase CD45 promotes onset and severity of IL-1β-mediated autoinflammatory osteomyelitis

Supporting information

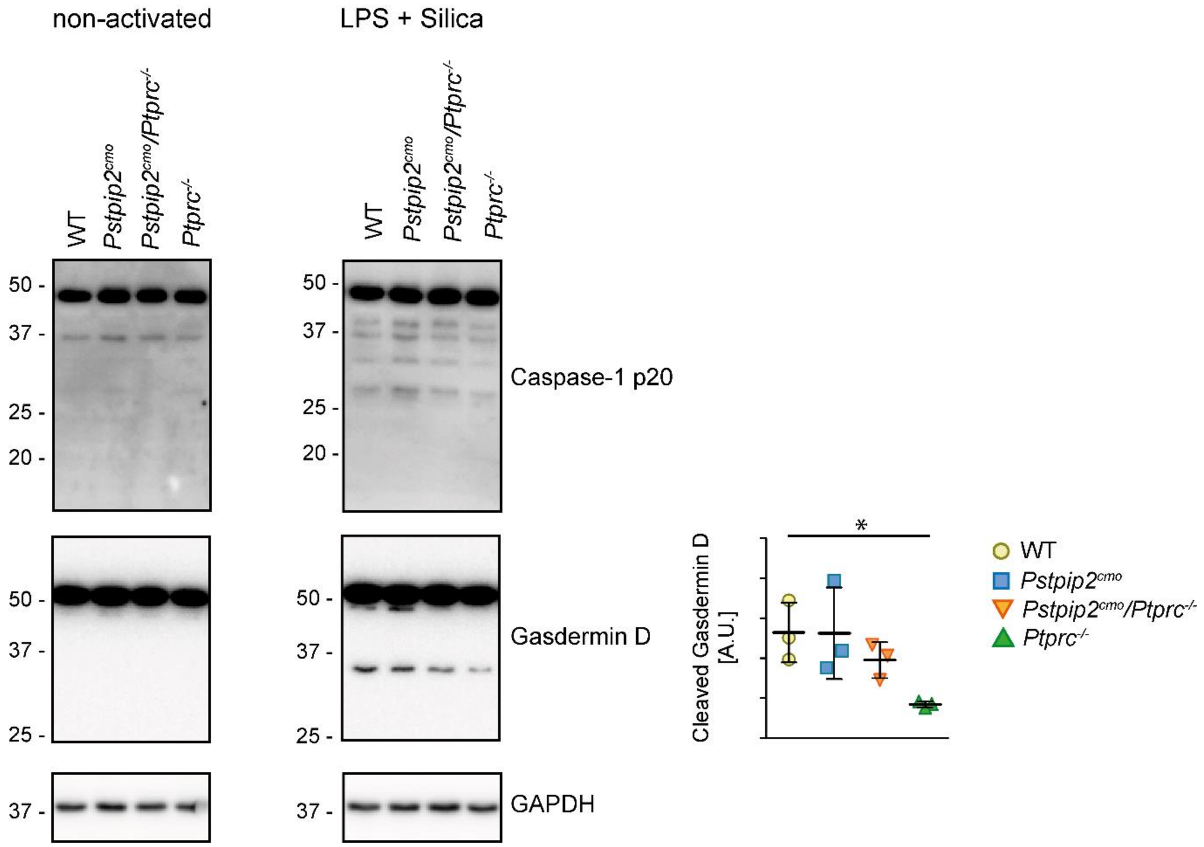

Figure S1. Inflammasome activity in neutrophils. Lysates of non-stimulated and LPS/silica stimulated neutrophils isolated from mice of indicated genotypes were analyzed by immunoblotting with antibodies to Caspase-1 p20 and Gasdermin D. GAPDH staining served as a loading control. Quantification of cleaved gasdermin D (p30) after normalization to GAPDH signal in activated samples from multiple experiments is shown on the right.
